# Distribution Map of Frost Resistance for Cement-Based Materials Based on Pore Structure Change

**DOI:** 10.3390/ma13112509

**Published:** 2020-05-31

**Authors:** Nguyen Xuan Quy, Takumi Noguchi, Seunghyun Na, Jihoon Kim, Yukio Hama

**Affiliations:** 1 Department of Civil Engineering and Architecture, Muroran Institute of Technology, Hokkaido 0508585, Japan; quynx@hau.edu.vn (N.X.Q.); 19096014@mmm.muroran-it.ac.jp (T.N.); bmjhun@mmm.muroran-it.ac.jp (J.K.); 2 Department of Civil Engineering, Hanoi Architectural University, Hanoi 100000, Vietnam; 3 Institute of Industrial Science, the University of Tokyo, 4-6-1 Komaba, Meguro-ku, Tokyo 153-8505, Japan; nash1122@naver.com

**Keywords:** frost resistance, pore structure, cement-based materials, mercury intrusion porosimetry, maturity, durability factor

## Abstract

This paper presents a prediction method and mathematical model based on experimental results for the change in pore structure of cement-based materials due to environmental conditions. It focuses on frost damage risk to cement-based materials such as mortar. Mortar specimens are prepared using water, ordinary Portland cement, and sand and the pore structure is evaluated using mercury intrusion porosimetry. New formulas are proposed to describe the relationship between the pore structure change and the modified maturity and to predict the durability factor. A quantitative prediction model is established from a modified maturity function considering the influences of environmental factors like temperature and relative humidity. With this model, the frost resistance of cement-based materials can be predicted based on weather data. Using the prediction model and climate data, a new distribution map of frost damage risk is created. It is found that summer weather significantly affects frost resistance, owing to the change in pore structure of cement-based mortar. The model provides a valuable tool for predicting frost damage risk based on weather data and is significant for further research.

## 1. Introduction

Frost damage is a serious problem for concrete structures in cold regions. It is widely recognized that cement-based materials in areas that experience cold temperatures are exposed to freeze–thaw cycles that can cause frost damage and reduce service lifetime [1,2]. As well as theories about frost damage, limited mathematical models and methods have been proposed to make reliable predictions of the frost resistance of cement-based materials during their lifetime [3,4,5,6]. However, while numerous investigations have focused on the frost resistance of cement-based materials [7,8,9,10,11], no previous literature has been published concerning a prediction model for a distribution map of frost resistance due to the change in pore structure.

Typical methods include the risk of frost damage and the ASTM (American Society for Testing and Materials) equivalent number of cycles (Cy_ASTM-sp_) are used for a distribution map of frost resistance. Hasegawa and Hon [12] calculated a value for frost damage risk from the temperature, solar radiation, winter precipitation, and snowmelt. Meanwhile, Cy_ASTM-sp_ determines the regional characteristics of frost damage of target cement-based materials by converting the freezing and thawing actions received per year from the temperature and environmental conditions into a number of freezing and thawing cycles in the standard of ASTM C-666 A method [13,14,15,16]. It is well known that the frost damage resistance of concrete depends on the pore structure [2,17,18]. In particular, the number of pores with a diameter of 40–2000 nm has a large effect on the frost damage resistance [19,20,21,22]. It is said that drying increases the pore volume of pores with a diameter of 40–2000 nm and decreases the frost resistance [23,24,25,26]. Thus, changes in pore structure are a necessary focus of research in frost resistance.

**Figure 1:**
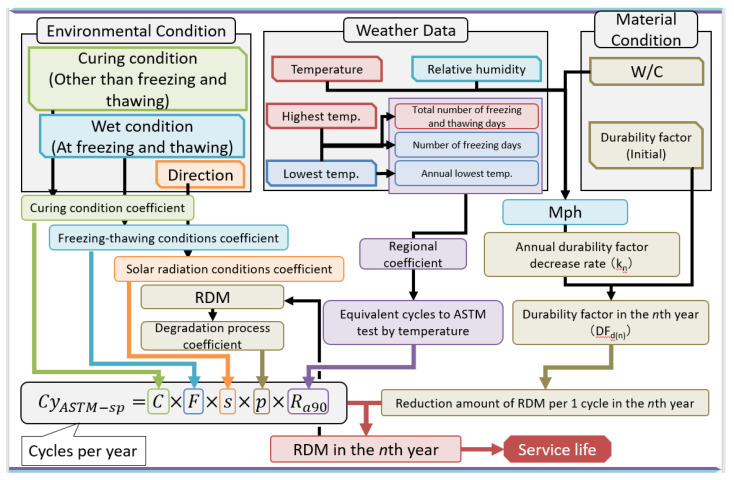
Overall flowchart for the calculation process.

The frost damage risk and Cy_ASTM-sp_, which are existing evaluation indexes of frost damage, evaluate only freeze–thaw actions in winter. However, these indicators cannot take into account the effects of reduced frost resistance due to summer drying. Both of these methods need to be improved. Since there is a correlation between frost damage resistance and the amount of pores with a diameter of 40–2000 nm, it is used to determine the service life of cement-based materials in terms of Cy_ASTM-sp_. Figure 1 shows a flowchart for calculation of the service life of cement-based materials, taking into account the changes in pore structure due to the environment.

The probability of occurrence of frost damage depends on environmental factors such as temperature and solar radiation and on the moisture content of cement-based materials [27,28,29,30,31,32,33,34,35]. Therefore, it is clear that the probability and risk of frost damage will vary from region to region, as illustrated by a distribution map of the risk of frost damage. Furthermore, it has been clarified that frost damage resistance decreases as result of changes in pore structure. This paper has confirmed that the pore structure changes depend on environmental conditions such as temperature and humidity. From this point of view, creating a distribution map of environmental indicators of frost damage and visualizing the regional characteristics of frost damage is very important.

Therefore, this study established a calculation method for combining functions of time, temperature, and humidity with Cy_ASTM-sp_, and examined the applicability of each meteorological property to environmental indicators of frost damage. The environmental factors are the curing conditions (other than freezing and thawing), the freezing and thawing conditions, the temperature and humidity as weather conditions, the water–cement ratio (W/C), and material conditions. The aim of this paper is to investigate the service life of cement-based materials affected by the change in pore structure due to environmental conditions. This will be accomplished by investigating those factors and the initial durability factor determining Cy_ASTM-sp_ (which is the number of freeze–thaw cycles received per year), ΔE_(n)_ (which is the decrease in relative dynamic elastic modulus per cycle) and determining the amount of decrease in relative dynamic elastic modulus per year. It is considered that the number of cycles (N_90_ and N_60_) at which the relative dynamic elastic modulus (E_d_) drops to 90% or 60% and the durability factor can be calculated from the volume of the 40–2000 nm diameter pores and the maturity function of time, temperature, and humidity. 

## 2. Materials and Methods

In this experiment, mortar specimens were prepared using Type I ordinary Portland cement (OPC) and natural sand. Natural sand with the maximum nominal size of 4.75 mm were used as fine aggregates in mortar. Fine aggregate has a specific gravity of 2.673 g/cm^3^, water absorption of 1.68% and the fineness modulus is 2.98. The chemical composition obtained by performing X-Ray Fluorescence (Malvern Panalytical Ltd, Malvern, UK) analysis and physical properties of the Type I ordinary Portland cement are shown in Table 1.

Twelve prismatic mortar samples of 40 × 40 × 160 mm^3^ were prepared with a water-to-cement ratio of 0.45 and a cement-to-sand ratio of 1:3 without any chemical admixture. In a mortar mixer, the cement, sand were first dry mixed until uniform and water was then added. The mixture was stirred evenly. The flow ability and the air content of fresh mortar were 189 mm and 5.3%, respectively. The mortar was cast, compacted via trowel rod with 25 standard blows. Then after, the mortar specimens were cured for 24 h at an ambient temperature of 20 ± 3 °C to achieve its setting. After demolding, the specimens were cut into cubes of 40 × 40 × 40 mm^3^ and subjected to initial curing.

Initial curing took place for 4 weeks at 20 °C in water. Then the specimens were immediately placed in secondary curing conditions at 20 °C, 35 °C, and 50 °C in various conditions of relative humidity (RH; 23%, 60%, 75%, and 100%) for a period of 26 weeks. After curing under each environmental condition, the samples were cut into 5 × 5 × 5 mm^3^ cubes, excluding the material within 1 cm of the surface and soaked in ethanol for 7 days to stop the hydration reaction of cement. This was then followed by drying through vacuum freeze dryer machine, pretreatment for 1 day.

Next, the pore structure of the mortar specimens was characterized using mercury intrusion porosimetry (MIP) according to Japanese Industrial Standards (JIS R 1655). MIP is a widely used technique for studying pore size distribution in cement-based materials. It applies Washburn’s formula [35,36]:

(1) $$r=-\frac{2\sigma_{m}\cos\theta_{m}}{P},$$

where P is the pressure, r is the capillary radius, θ_m_ is the contact angle between the mercury and the surface of the solid material tested, and σ_m_ is the surface tension of the mercury. The mercury intrusion porosimeter (PoreMaster 33, Quantachrome Instruments, Boynton Beach, FL, USA) can achieve hydraulic pump-generated pressures of 0–228 MPa.

## 3. Model Development

### 3.1. Results for Pore Structure Change Due to Environmental Conditions

**Figure 2:**
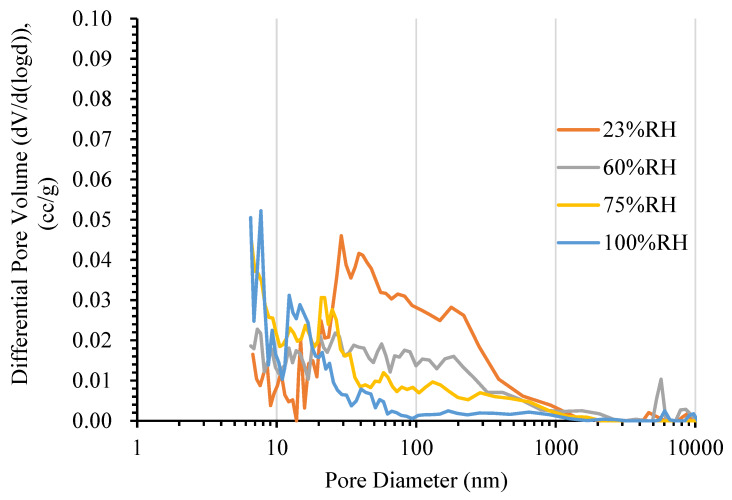
The differential pore size distribution of mortar at 20 °C.

To investigate the effects of environmental conditions on pore structure changes of cement-based materials, MIP tests were carried out on the mortar samples in different conditions. The pore size distribution obtained from the MIP tests was used to investigate the change in pore structure. In this study, the influence of environmental conditions on the change in pore structure is clear. The experimental results show that the change in pore structure depends greatly on temperature and humidity. As can be seen in Figure 2, the more the humidity increases (from 23% to 100% RH), the smaller the critical pore entry diameter tends to be (29 nm down to 7 nm). Changes of the critical pore entry diameter of mortar can be explained by the loose packing of the hydration products—calcium silicate hydrate (C-S-H) gel will be converted to low-density C-S-H at very low RH as reported by Wu et al. [37].

**Figure 3:**
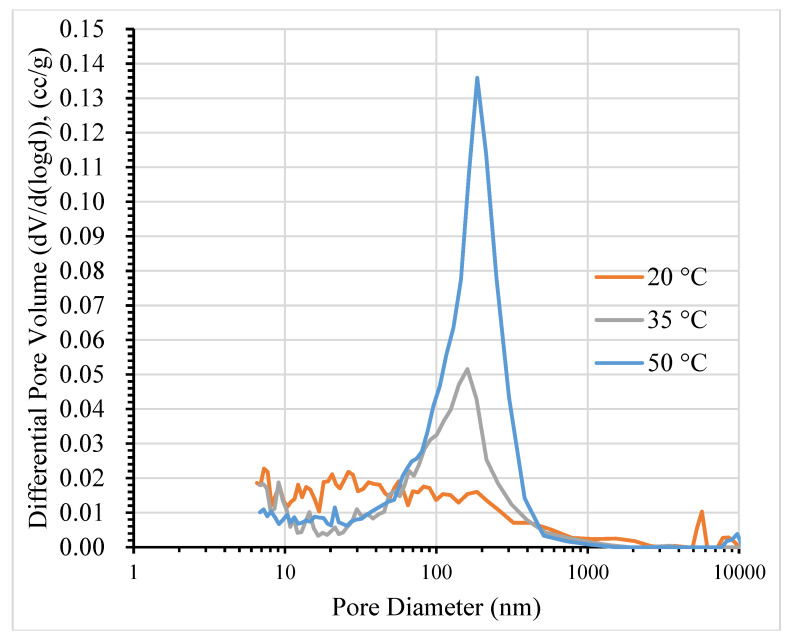
The differential pore size distribution of mortar at 60% RH.

In addition, it can be clearly seen that, as the temperature increases, the critical pore entry diameter also increases, as in Figure 3. The highest derivative peak in the differential pore size distribution increased sharply when the temperature reached 50 °C. The results in Figure 3 also give evidence for the coarsening of the pore structure, in good agreement with the findings of Suwanmaneechot et al. [38] and Jennings [39]. The main cause of coarsening of the pore structure is the nanostructure change of C-S-H at the drying temperature, according to Aono et al. [23].

As mentioned above, it is clear that the pore structure change is significantly influenced by environmental conditions. Therefore, in this study, practical formulas such as the modified maturity as a function of time, temperature, and humidity based on the presented findings [40,41] are formulated by:

(2) $$\begin{array}{c} M_{\mathrm{ph}}=\overset{n}{\underset{t=1}{\sum }}(\theta_{d,t}-D_{t})\left(H_{t}-\varphi_{d,t}\right)\Delta t,\\ D_{t}=16,\;\mathrm{and}\\ H_{t}=0.35\left(\frac{W}{C}\right)+0.6392, \end{array}$$

where M_ph_ is the modified maturity function using time, temperature, and humidity (°C·days); θ_(d,t)_ is the curing temperature (°C); D_t_ is the datum temperature at which an increase in volume of 40–2000 nm diameter pores does not occur (°C); H_t_ is the RH of the curing environment (%); and φ_(d,t)_ is the datum humidity at which an increase in pore volume does not occur.

According to previous research [41], the change in the volume of 40–2000 nm diameter pores that corresponds to a specific temperature–humidity environment and water-to-cement ratio is obtained from:

(3) $$\begin{array}{c} \ln\left(\frac{{\mathrm{PV}}_{d}}{{\mathrm{PV}}_{i}}\right)=w\sqrt{M_{\mathrm{ph}}}\left(0\leq \sqrt{M_{\mathrm{ph}}}<\sqrt{b}\right)\;\mathrm{and}\\ \ln\left(\frac{{\mathrm{PV}}_{d}}{{\mathrm{PV}}_{i}}\right)=m\;\left(\sqrt{M_{\mathrm{ph}}}\geq \sqrt{b}\right), \end{array}$$

where w is the slope of a straight line to the upper limit, m is the upper limit of the pore volume, and b is an empirical constant); PV_i_ is the volume of 40–2000 nm diameter pores after initial curing (cc/g); and PV_d_ is the volume of 40–2000 nm diameter pores after drying (cc/g).

The relationship between drying conditions and the change in pore structure is calculated with Equation (3) using the parameters obtained from the exposure test results. The modified maturity function in Equation (2) and the pore volume in Equation (3) will be used in Section 3.2.

### 3.2. Model for Predicting Frost Resistance Based on Pore Structure Change

Kamada et al. [19] found that the relationship between the volume of 40–2000 nm diameter pores, the air-content-to-paste ratio, and the durability factor is represented by:

(4) $$\log\left(\mathrm{DF}\right)=-0.317-1.209\log\left(\mathrm{PV}\right)+1.799\frac{A}{P},$$

where DF is the durability factor, PV is the volume of 40–2000 nm diameter pores (cc/g), and A/P is the air-content-to-paste ratio (%).

The ratio of the durability factors before and after the change in pore structure calculated from Equation (4) and combined with Equations (2) and (3) can be expressed as:

(5) $$\frac{{\mathrm{DF}}_{d}}{{\mathrm{DF}}_{i}}={\left(\frac{{\mathrm{PV}}_{d}}{{\mathrm{PV}}_{i}}\right)}^{-1.209}={\left(\exp\left(w\sqrt{M_{\mathrm{ph}}}\right)\right)}^{-1.209},$$

where DF_d_ is the durability factor after drying and DF_i_ is the durability factor at initial conditions (water curing at 20 °C for 4 weeks is the standard).

Furthermore, this study established a prediction model that indicates the service life due to frost damage using the Cy_ASTM-sp_ and the modified maturity function. It uses the modified maturity to take into account the decrease in frost damage resistance caused by summer drying. Therefore, a forecast of frost damage must also consider the change of pore structure during summer. This means that the frost damage of cement-based materials predicted by the modified maturity function is required, in addition to that determined by region in winter from Cy_ASTM-sp_, in order to calculate the service life. The service life is determined by finding the maximum number of years in which the relative dynamic elastic modulus does not fall below 60%. Cy_ASTM-sp_ is calculated using the coefficients shown in Table 2, using:

(6) $$\begin{array}{c} {\mathrm{Cy}}_{\mathrm{ASTM}-\mathrm{sp}}=C\times F\times s\times p\times R_{a90},\\ R_{a90}=4.2T-5.4,\;\mathrm{and}\\ T=-t_{a\;\min}\left(1-\frac{D_{f}}{D_{w}}\right), \end{array}$$

where Cy_ASTM-sp_ is the number of ASTM equivalent cycles (cycle/year), C is the coefficient for the curing conditions, F is the coefficient for the freeze–thaw conditions, s is the coefficient for the sunlight conditions, p is the coefficient for the degradation process, R_a90_ is the effective number of Cy_ASTM-sp_ cycles in air temperature (cycles/year), T is a regional coefficient, t_(a min)_ is the annual minimum temperature (°C), D_f_ is the total number of freezing and thawing days, and D_w_ is the number of freezing days.

**Figure 4:**
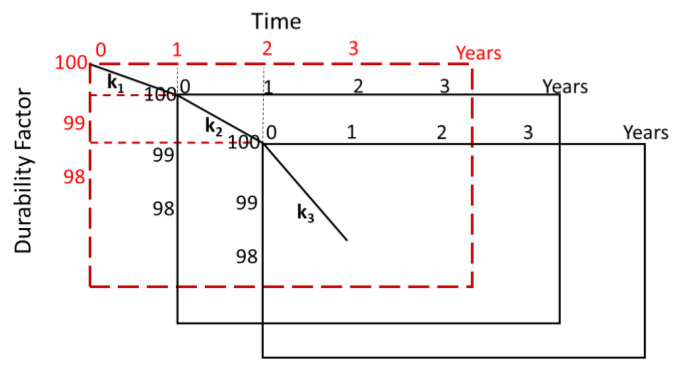
Conceptual diagram of the relationship between durability factor and elapsed years.

In an actual environment, pore structure change causes a decreased durability factor, so the relative dynamic elastic modulus of cement-based materials cannot be represented by a simple straight line. Therefore, from the ratio of the durability factors, the decrease in the durability factor for each year is calculated. Because the maturity function varies every year, the evaluation of the durability factor changes year by year (as the first year, second year, …, n^th^ year). Furthermore, as shown in Figure 4, the durability factor changes with aging and is calculated as a ratio, k, of that of the previous year. Since the initial durability factor is maintained, the axis deviates as it is re-evaluated the following year. Therefore, considering a ratio k for each year, it is possible to predict a future value, as shown by the broken line in Figure 4.

The durability factor in the n^th^ year is represented using k_(n)_ as follows:

(7) $${\mathrm{DF}}_{d\left(n\right)}={\mathrm{DF}}_{d\left(n-1\right)}\times k_{n}$$

and the ratio k_(n)_ the n^th^ year to that of the previous year in is expressed by:

(8) $$k_{n}=\frac{{\mathrm{DF}}_{d\left(n\right)}/{\mathrm{DF}}_{i}}{{\mathrm{DF}}_{d\left(n-1\right)}/{\mathrm{DF}}_{i}}.$$

Next, the amount of reduction of the relative dynamic elastic modulus for each cycle is calculated. With respect to the durability factor DF, since the calculation method is different when DF ≥ 60 and when DF < 60, the amounts of reduction are respectively calculated by:

(9) $$\Delta E_{\left(n\right)}=\frac{100-{\mathrm{DF}}_{d\left(n-1\right)}}{300}\;\left(\mathrm{DF}\geq 60\right)\;\mathrm{and}$$

(10) $$\Delta E_{\left(n\right)}=\frac{100-P}{N}\;(\mathrm{DF}<60),$$

where ΔE_(n)_ represents the amount of decrease of the relative dynamic elastic modulus in the n^th^ year.

**Figure 5:**
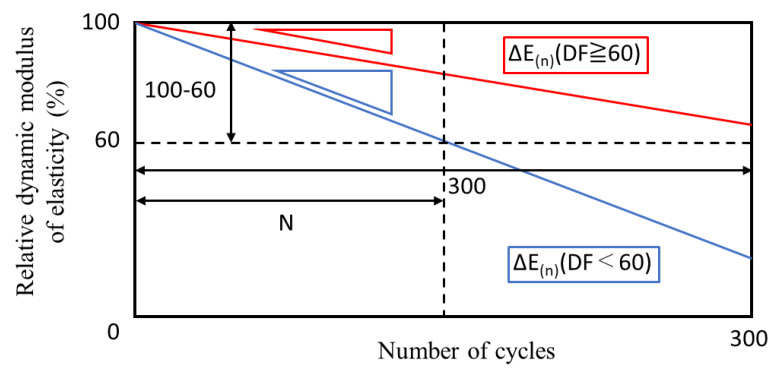
Relative dynamic elastic modulus decreasing per cycle.

As shown in Figure 5, ΔE_(n)_ represents the slope of the relative dynamic elastic modulus with respect to the number of cycles, and this is different when DF ≥ 60 and when DF < 60. Moreover, considering the process of actual frost damage, it seems that the relative dynamic elastic modulus does not decrease with a certain value as shown in Figure 5. In this study, however, cement-based materials are supposed to deteriorate constantly every cycle in order to simplify calculation.

Equation (10) can be rewritten to Equation (12) by substituting Equation (11) into Equation (10):

(11) $$N=\frac{M\cdot {\mathrm{DF}}_{\left(n-1\right)}}{P}\;\mathrm{and}$$

(12) $$\Delta E_{\left(n\right)}=\frac{100-P}{\frac{M\cdot {\mathrm{DF}}_{\left(n-1\right)}}{P}}=\frac{100-60}{\frac{300\cdot {\mathrm{DF}}_{\left(n-1\right)}}{60}}=\frac{8}{{\mathrm{DF}}_{\left(n-1\right)}},$$

where k_n_ represents the ratio of the durability factor to that of the previous year in the n^th^ year, and DF_d(n)_ represents the durability factor in the n^th^ year.

Using the calculated amount of decrease in relative dynamic elastic modulus per cycle and Cy_ASTM-sp_, the amount of decrease in the relative dynamic elastic modulus in each year is calculated. The relative dynamic elastic modulus in each year is calculated from the amount of decrease in the relative dynamic elastic modulus by:

(13) $$\Delta {\mathrm{RDM}}_{\left(n\right)}={\mathrm{Cy}}_{\mathrm{ASTM}-\mathrm{sp}}\times \Delta E_{\left(n\right)}$$

(14) $${\mathrm{RDM}}_{\left(n\right)}={\mathrm{RDM}}_{\left(n-1\right)}-\Delta {\mathrm{RDM}}_{\left(n\right)}$$

By integrating the relative dynamic elastic modulus, which decreases every year in real environments, it is possible to express the relationship between elapsed years and relative dynamic elastic modulus:

(15) $${\mathrm{RDM}}_{\left(n\right)}=100-\sum_{j=1}^{n}{\mathrm{Cy}}_{\mathrm{ASTM}-\mathrm{sp}}\cdot \Delta E_{\left(j\right)},$$

where RDM_(n)_ represents the relative dynamic elastic modulus in the n^th^ year. The service life is the number of years until the relative dynamic elastic modulus falls below 60%.

## 4. Model Validation and Discussion: An Application Related to Japan’s Climate

Japan has four distinct seasons with climates varying from the Hokkaido to Honshu regions. Hokkaido has warm summers and very cold winters with heavy snow and it frequently observes winter temperatures as low as −20 °C. Meanwhile, Honshu has hot and humid summers and cold winters and it sometimes experiences summer temperatures of 35 °C or above. 

As above, a prediction model that calculates the service life with frost damage after taking into account the effects of drying is proposed. The applicability of the meteorological statistical data used in the calculation is also examined. The greatest advantage of this model is that the climate data (temperature and relative humidity) of each country around the world can be used for this model. In this study, the frost resistance prediction of the proposed model is based on Japanese meteorological data. It uses the latest meteorological data to create a new distribution map that evaluates the regional nature of frost damage. All the regional climate data used in the calculation model are according to the Japan Meteorological Agency [42,43]. 

The service life is determined by finding the maximum number of years in which the relative dynamic elastic modulus does not fall below 60%. However, even when comparing the distribution maps of weather indicators of frost damage taking into consideration the risk of frost damage and the effects of drying, it is difficult to see the effects of drying purely because the evaluation methods differ greatly. Therefore, the effect of drying is evaluated by creating a distribution map of Cy_ASTM-sp_. By creating a new distribution map of the frost damage, the effects of drying on frost damage can be visualized and the regional nature of frost damage evaluated. 

The Cy_ASTM-sp_ and modified maturity values of each region in Japan are presented in Table 3. For the frost damage environment index considering Cy_ASTM-sp_ and the effect of drying, the environmental conditions used were the solar radiation conditions on the north side, the curing and drying conditions at 20 °C, and the freezing and thawing conditions in air and frozen water. In addition, N_90_ was set to 300 and N_60_ was set to 900 as conditions for calculating the service life based on Cy_ASTM-sp_. The frost damage environment index taking into account the effects of drying was calculated under two conditions, 60 and 90 DF_i_, with W/C 45%. In Table 3, an interesting trend can be observed. Depending on the weather data of each city, the range of modified maturity values varies from 5.3 to 81.9 °C·day in Hokkaido and from 14 to 218.7 °C·day in Honshu. It can be clearly seen that the modified maturity values in cold regions are smaller than those in hot regions. Therefore, the effect of summer drying temperature can be expressed by the modified maturity index.

**Figure 6:**
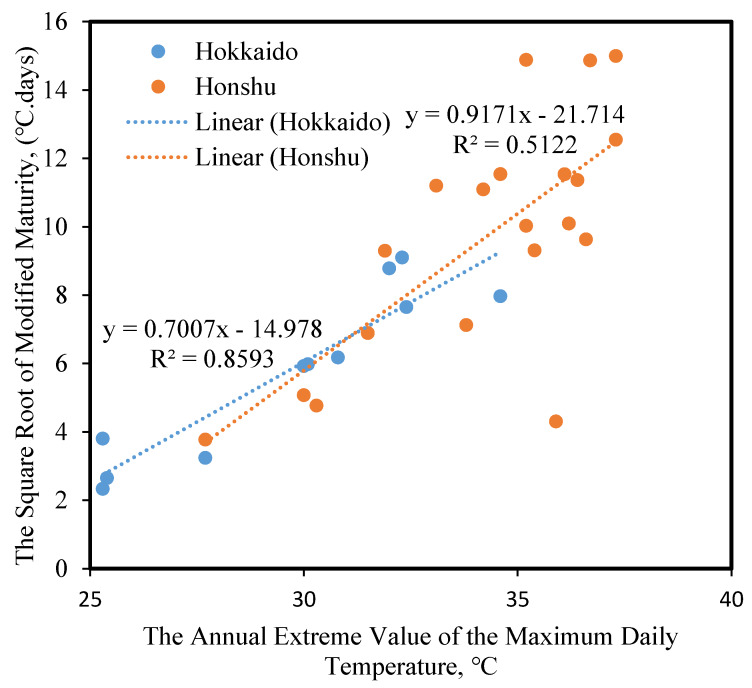
Relationship between the annual extreme value of daily maximum temperature and the modified maturity in each region in Japan.

Figure 6 shows the relationship between the annual extreme value of daily maximum temperature and the modified maturity in each region. The modified maturity tends to increase as the daily maximum temperature increases, not only in Hokkaido but also in Honshu. This finding shows that the modified maturity given in Equation (2) can be used with confidence. In particular, the relationship, in the case of the Hokkaido region, exhibits a higher correlation than in the case of the Honshu region. The trends in Figure 6 suggest that the modified maturity is affected considerably and directly by the summer drying temperature. Meanwhile, the winter temperature is too low in the Hokkaido region and is outside of the calculation of modified maturity. This is probably because, in the Honshu region, the short freeze–thaw period in winter and the large differences in relative humidity from region to region are the main cause of variation.

**Figure 7:**
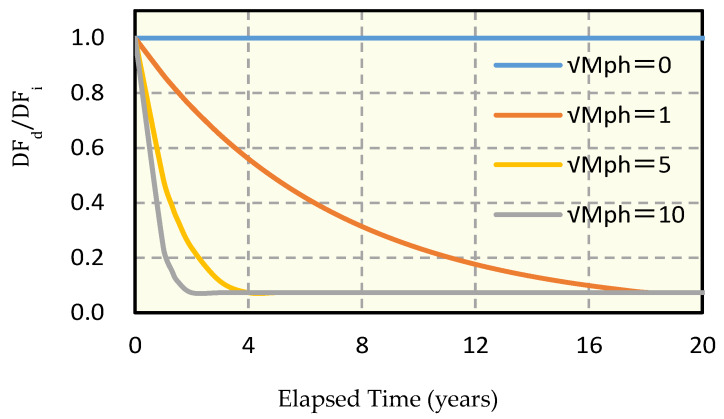
Degradation of durability factor for different modified maturities.

The effect of the modified maturity on the ratio of the durability factors (DF_d_/DF_i_) is shown in Figure 7, which gives the relationship between the durability factor for different modified maturities and the elapsed years before calculating the service lifetime. It can be seen from Figure 7 that the durability factor ratio decreases gradually as the square root of the modified maturity increases. Interestingly, both DF_d_ (the durability factor after drying) and the modified maturity are the factors that clearly express the effect of drying due to the weather conditions. In addition, even assuming that the weather in winter is the same, the modified maturity increases as a result of the effects of the summer drying temperature and the frost damage resistance may decrease. Based on this finding, the relationship between the modified maturity and the durability factor may be considered as the first meteorological index of the effect of summer drying conditions on frost damage deterioration.

**Figure 8:**
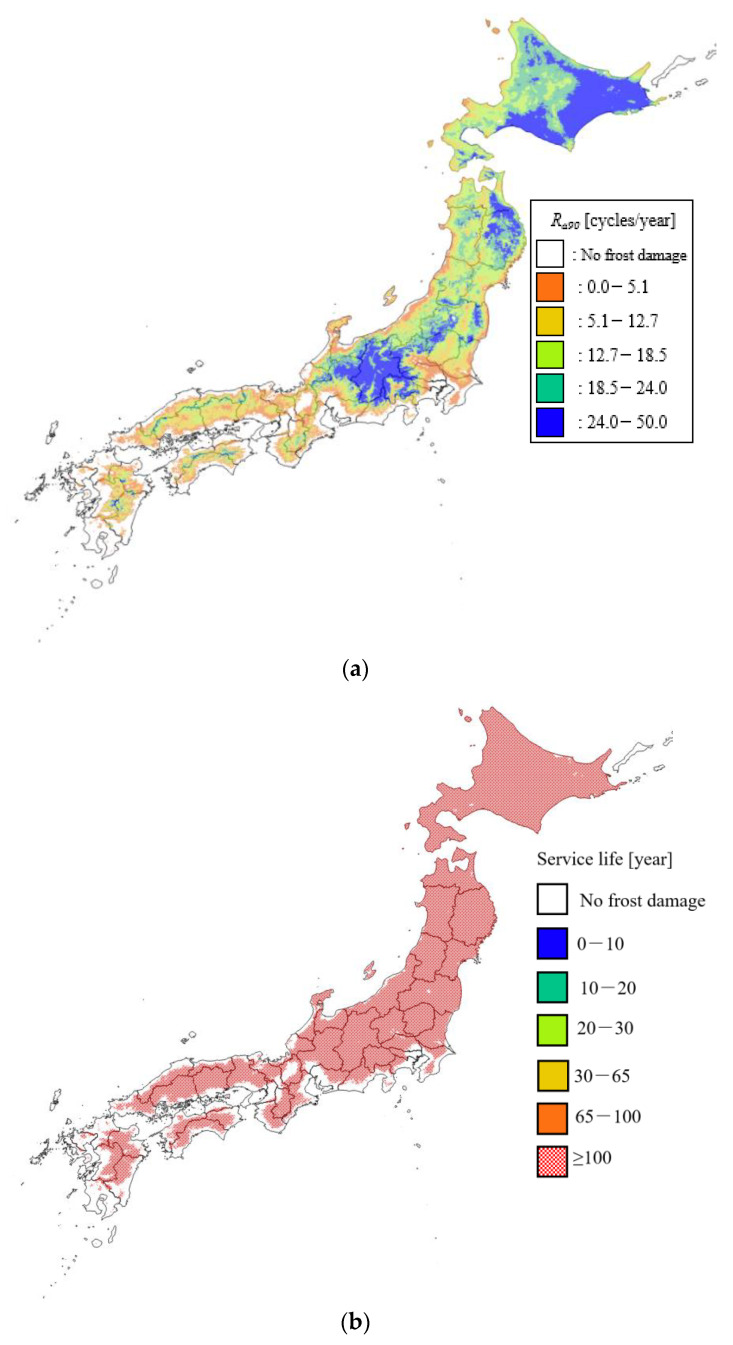
Distribution maps of: (**a**) R_a90_ based on temperature and (**b**) service life based on Cy_ASTM-sp_. Solar radiation conditions: north side; curing conditions: 20 °C drying; freezing and thawing conditions: thawing in air; number of cycles at which relative dynamic elastic modulus is 90%: 300; and number of cycles at which the relative dynamic elastic modulus becomes 90%: 900.

As mentioned previously in Section 3.2, Cy_ASTM-sp_ is used to estimate the effect of freezing temperature on frost damage and the effective number of freeze–thaw cycles, which is corrected to an ASTM equivalent number of cycles. Cy_ASTM-sp_ was calculated based on previous work [44], but with updated current climate data, and a distribution map was created. Figure 8a shows a distribution map of R_a90_ according to the temperature and Figure 8b shows a distribution map of the service life according to the Cy_ASTM-sp_ method using updated climate data. It can be seen in Figure 8a that R_a90_ is high in the eastern Hokkaido, northern Iwate, and Nagano Prefectures. The areas that are evaluated as experiencing frost damage are mainly Chiba Prefecture on the Pacific side, along the west coast of Niigata Prefecture, and some inland areas of Kyushu. Compared with the frost damage risk in Japan according to Hasegawa and Hon [12], the areas where frost damage does not occur (that is, the risk of frost damage is 0 and the risk of frost damage is not covered by R_a90_) are very small, and the interior of Hokkaido is very small. The difference is a relatively small value. With regard to frost damage risk, even if some freezing and thawing has occurred, it is judged that frost damage will not occur in areas where the frost damage risk is below a certain value. On the other hand, in the R_a90_ distribution map, only areas where t_amin_ is approximately −1.3 °C or higher and water in concrete is very unlikely to freeze are treated as exempt from frost damage. As a result, R_a90_ was stricter in the evaluation of the presence of frost damage, and, even in Honshu, most of the area except the coastal area indicated the presence of frost damage. The inland areas of Hokkaido have high altitude and low temperature, so the number of freezing days increases, the regional coefficient T becomes smaller, and R_a90_ is lower than in surrounding areas such as eastern Hokkaido.

From the above, it becomes clear that the evaluation of the presence or absence of frost damage by Cy_ASTM-sp_ was more severe than the degree of frost damage risk, and that it was easily affected by the duration of freezing. In addition, the service life of cement-based materials (assuming a durability factor of 90) was over 100 years in all regions including Hokkaido.

**Figure 9:**
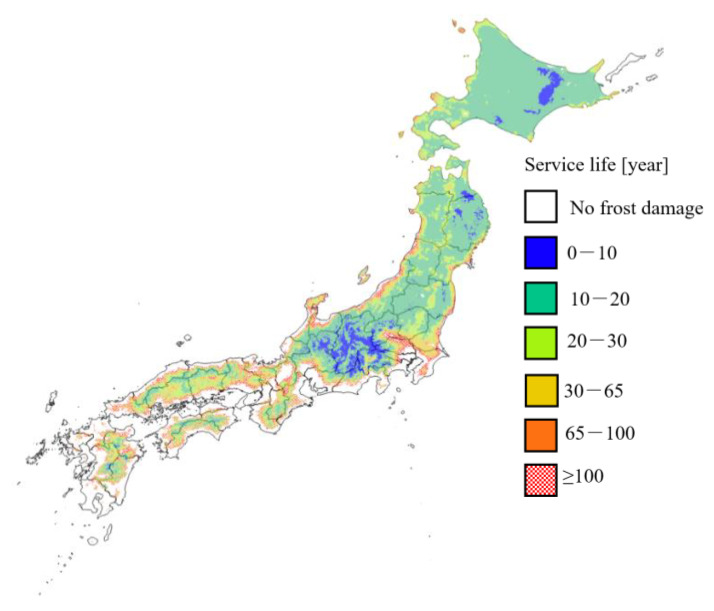
Distribution map of service life considering the effect of pore structure change due to drying with an initial durability factor of 90. Solar radiation conditions: north side; curing conditions: 20 °C drying; freezing and thawing conditions: thawing in air; and water-to-cement ratio: 0.45.

Figure 9 shows the distribution of service life considering the effect of pore structure change due to drying of concrete, assuming a durability factor of 90. This service life is 30 years or less in many areas subject to frost damage, and is significantly reduced compared to the service life based on Cy_ASTM-sp_, which does not consider the effects of drying. In Honshu, where the temperature is higher than in Hokkaido, the modified maturity is large, and the rate of frost damage resistance declines quickly. As a result, the service life in the mountainous area of Honshu is less than 20 years, which is almost the same as in the whole area of Hokkaido. Considering the decrease in frost damage resistance due to drying, there is a wide range of risk of frost damage in Honshu as well as in Hokkaido.

**Figure 10:**
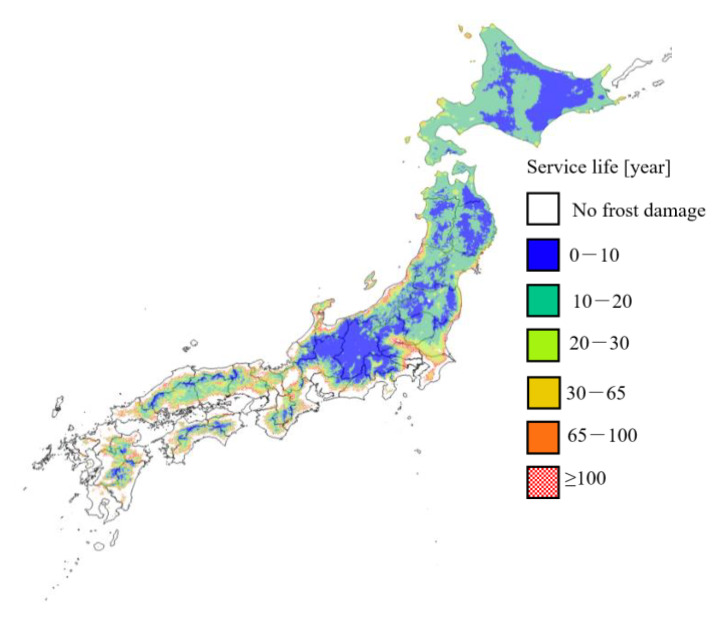
Distribution map of service life considering the effect of pore structure change due to drying with an initial durability factor of 60. Solar radiation conditions: north side; curing conditions: 20 °C drying; freezing and thawing conditions: thawing in air; and water-to-cement ratio: 0.45.

Figure 10 shows the distribution of service life considering the effect of pore structure change due to drying of mortar assuming a durability factor of 60. With this, the areas with a service life of less than 10 years have expanded, mainly in eastern Hokkaido, the mountainous areas of the Tohoku region, the entire area of Nagano Prefecture, and the northern part of Gifu Prefecture. There is no significant change for regions that are older than a year. It is considered that these areas maintain a service life of more than 100 years even if the durability factor is lowered because R_a90_ is close to but not zero. In most regions where the durability factor is 90 and the service life is 20–30 years, the service life is reduced to 10–20 years and to almost 10 years when the durability factor is 60. From this, it can be said that, when the durability factor is 60, the risks of frost damage in Honshu and Hokkaido are more equal than in the case where the durability factor is 90.

In addition, it became clear that, by considering drying, the deterioration progressed rapidly and the service life was greatly reduced. Furthermore, it was found that, even in temperate regions, the risk of frost damage when considering drying may be equivalent to that in cold regions, especially in construction materials with a low durability factor. In particular, by considering the effects of drying, it is important to note that the risk of frost damage in Honshu and Kyushu can be equal to that in Hokkaido. When comparing the conventional Cy_ASTM-sp_ method and the service life, even in areas where the influence of the winter seasons is small and the risk of frost damage deterioration was underestimated, it turned out that the service life was shorter when considering the influence of summer.

From the distribution map of the service life of cement-based materials assuming a durability factor of 90, it became clear that the progress of deterioration rapidly increased as a result of drying, and the service life decreased significantly. Furthermore, it was confirmed that the risk of frost damage in some parts of Honshu was the same as that in Hokkaido, and this was more pronounced as the initial durability factor was lower. By evaluating the regional characteristics of frost damage based on the distribution map, as a means of utilizing the new environment index for frost damage, it is possible to develop maintenance plans such as proactive repair plans and the selection of structures that should be subject to intensive degradation investigations. It is proposed to use it for compounding planning considering the repair cycle. 

## 5. Conclusions

Based on the results presented in this study, several conclusions can be drawn:

- It was confirmed that the change in pore structure due to drying at high temperature in the summer greatly decreased the durability factor of cement-based materials, as the annual maturity value based on time, temperature, and humidity was larger.
- A distribution map of frost damage risk was created using meteorological data for the frost damage deterioration of mortar. The service life becomes shorter when considering the influence of summer drying. From the distribution map of the service life assuming the same durability index, it became clear that the deterioration progressed rapidly and the service life was significantly reduced by considering the drying.
- It was found that, even in warm regions, the risk of frost damage could be the same as in cold regions when taking into account the effects of drying, and that the tendency was more pronounced for concrete with a low durability factor.
- The environment index and its distribution map established and created by this research can be used for regional evaluation as well as proposed utilization for applications such as management that contribute to extending the life of buildings.
- The climate data of each country around the world can be used for prediction model, therefore, future work is also needed to investigate the effect of various climate types such as tropical, temperate and polar on prediction model and develop a software using the results of this study.

## Figures and Tables

**Table 1:** Chemical compositions of ordinary Portland cement.

Items	Unit	Testing Value
Specific gravity	g/cm^3^	3.161
Specific surface area	cm^2^/g	3368
MgO	%	2.0
SO_3_	%	1.8
SiO_2_	%	22.6
Al_2_O_3_	%	6.2
Fe_2_O_3_	%	3.1
CaO	%	62.3
LOI	%	0.68

**Table 2:** Coefficients for the Cy_ASTM-sp_ formula.

ASTM Equivalent Cycles, Cy_ASTM-sp_ = C.F.s.p.R_a90_			Process of Frost Damage	
			Signal 100% ≥ E_d_ > 90%	Clear 90% ≥ E_d_ > 60%
Degradation coefficient (p)			1.00	1.64
Condition Coefficients	Solar radiation (s)	North face	1.00	1.00
		South face	1.45	1.45
	Curing and drying (C) C	In water	1.00	1.00
		In air	0.66	1.41
		Drying at 20 °C	0.26	0.80
		Drying at 30 °C	0.14	0.45
	Freeze–thaw (F)	In water	1.00	1.00
		Freezing in air and thawing in water	0.21	0.23

**Table 3:** Cy_ASTM-sp_ and modified maturity values of each region in Japan.

Region/City		Cy_ASTM-sp_		Modified Maturity M_ph_ (°C·day)
		Cy_ASTM-90_	Cy_ASTM-60_	
Hokkaido	Wakkanai	0.3	1.4	14.2
	Kitami	0.6	3.2	37.7
	Asahikawa	1.1	6.2	81.9
	Abashiri	0.8	4.2	35.3
	Sapporo	0.9	4.8	76.2
	Obihiro	1.7	9.3	62.8
	Nemuro	0.7	3.8	6.9
	Suttsu	0.5	3.0	34.6
	Muroran	0.6	3.3	10.3
	Urakawa	1.2	6.9	5.3
	Hakodate	1.3	7.0	57.7
Honshu	Aomori	0.7	4.1	71.0
	Hachinohe	0.8	4.5	50.1
	Akita	0.4	2.0	100.7
	Morioka	1.1	6.2	85.4
	Miyako	0.7	4.1	46.8
	Sendai	0.2	1.2	85.6
	Yamagata	0.6	3.3	131.6
	Fukushima	0.2	1.2	155.8
	Tochigi	1.3	7.0	25.4
	Utsunomiya	0.4	2.2	133.5
	Karuizawa	1.9	10.5	22.4
	Matsumoto	1.1	5.8	154.0
	Kofu	0.3	1.7	218.7
	Takayama	1.1	6.0	119.1
	Okayama	0.2	1.2	158.5
	Asozan	0.9	5.1	14.0
	Yufuin	0.4	2.0	107.3
